# Personality and Genetics Correlations to Risk-Taking Using Quantum Decision Theory in Balloon Analogue Risk Tasks

**DOI:** 10.7759/cureus.9923

**Published:** 2020-08-21

**Authors:** Vincent A Weidlich

**Affiliations:** 1 Psychology, Northumbria University, Newcastle upon Tyne, GBR

**Keywords:** psychology, genetics, behavioral science, quantum, prediction, risk-taking

## Abstract

Genetics and personality can be determined and assessed in individuals, which can be used to predict behaviours in large groups and possibly individually. This report will describe how to use quantum decision theory (QDT) to predict these behaviours. Genetics and personality correlations to risk-taking using QDT in balloon analogue risk tasks (BART) will be covered in this report. The areas of theory covered will be BART, QDT, QDT in BART, personality correlations to risk-taking in BART, genetic correlations to risk-taking in BART, the models used in these theories and a presentation of new models to use these theories together.

This article reviews many other primary research articles, which analyses the correlation between genetics/personality and risk-taking behaviour in BART. This report provides models that use impulsivity, venturesomeness, and genetic traits with QDT, to probabilistically predict decisions in risk-taking behaviour.

## Introduction and background

This review article will focus on the use of quantum decision theory (QDT), along with personality traits and genetic traits, in order to predict individual and group behaviour. The aim of this research is to better understand how to predict individual and group behaviour decision processes, as well as to create models to probabilistically predict these decisions. Strong correlations between specific genetic traits and risk-taking have been found, as well as strong correlations between specific personality traits and risk-taking. Research has shown that QDT can be used to probabilistically predict decisions using certain models. These prediction models can be used for creating individual and group profiles for automated systems. Existing research in QDT, personality traits correlated to risk-taking in balloon analogue risk tasks (BART) studies and genetic traits correlated to risk-taking in BART studies will be used to be built upon, to develop new prediction models.

## Review

QDT is a recently developed theory of decision-making based on the mathematics of Hilbert spaces, a framework known in physics for its application to quantum mechanics [1]. In quantum mechanics, associated with one event, there are rational and irrational parts to its probability. QDT takes this idea and applies it to decision making, where the utility factor is rational, and the attractiveness factor is irrational. The utility factor is rational because normal logic can be used to understand it. The attractiveness factor is irrational because it does not use this normal logic and can be viewed as illogical. The $f(pi_n)$ in the model corresponds to the classical part of the probability and the $q(pi_n)$ in the model corresponds to the quantum ‘weirdness’ associated with the probability.

The ways in which personality and genetic correlations in BART studies can be used to create a risk-taking coefficient, which QDT can use to make probabilistic predictions for choosing between decisions will be examined. The mathematical structure of quantum physics can be used as inspiration for many applications apart from atoms, just as calculus is used for many applications apart from planetary movement. Haven and Khrennikov [2] describe the psyche of an individual as being represented by a state vector, denoted as |P 〉 whose elements are all possible states of an individual’s psyche. Psychology, sociology, economics and other subjects can use the maths and inspiration of quantum physics, to analyse, predict and much more.

Yukalov [3] suggested that the mathematical structure of QDT is common for both decision theory as well as for quantum measurements, which has been achieved by generalizing the von Neumann theory [4] of quantum measurements to the treatment of inconclusive measurements and composite events represented by noncommutative operators. John von Neumann presented a measurement upon a physical system by a self-adjoint operator on that Hilbert space termed an "observable.” In quantum physics, a measurement is the testing or manipulation of a physical system in order to yield a numerical result. The predictions that quantum physics makes are in general probabilistic. A self-adjoint operator or Hermitian operator on a finite-dimensional complex vector space V with inner product {.,.} is a linear map A (from V to itself) that is its own adjoint for all vectors v and w. A Hilbert space is an abstract vector space possessing the structure of an inner product between two states that gives you an amplitude, and the square of that corresponds to a probability.

The BART is a computerized measure of risk-taking behaviour. The BART models real-world risk behaviour through the conceptual frame of balancing the potential for reward versus loss. In the task, the participant is presented with a balloon and offered the chance to earn money by pumping the balloon up by clicking a button. Each click causes the balloon to incrementally inflate and money to be added to a counter up until some threshold, at which point the balloon is overinflated and explodes. When the balloon explodes, the participant loses any potential winnings (BART instrument) [4].

QDT can be used to predict decisions in BART. The attractiveness factor and utility factor will be part of the prediction method.

The attractiveness factor used in the research 

$&alpha;_n =&sum;_i x_i 10^{p(x_i)}$

$&alpha;$ = the attractiveness factor

$&sum;_i$ = the sum of all possible outcomes, £1 or £10 

$x_i$ is either £1 or £10 

$10^{p(x_i)}$, $p$ = the probability 

The attractiveness factor is a quantity that encompasses people’s irrationality in decision making. The expected winning informs the rational side of decision making. The attractiveness factor informs the irrational side of decision making. Both are functions of expected winnings and associated probabilities. The change of values can encompass how someone sees the probabilities. For example, if it is a million-pound jackpot, the amount will be more attractive.

Let us start with a set of outcomes, winning 1 or 10 pounds. The difference of a set of outcomes and a lottery is that we associate a probability with those outcomes. With each outcome, there will be an associated probability. 

X = set of outcomes

$L_n$ = the lottery

$u$ = the utility of that outcome

$U$ = the sum of all utilities weighted with the probability of each outcome

$p_n$ = the probability associated to that particular outcome

$i$ = the label for different outcomes

$N$ = the number of actual outcomes

$n$ = label of lottery

$X_n={x_i:i=1,2,&hellip;N_n}$

$L_n={x_i,p_n (x_i ):i=1,2,&hellip;N_n}$

$u(x_i)=x_i$

$U(L_n)=\SigmaU(x_i )p(x_i)$

$=&sum;_i x_i p_n (x_i)$

Utility factor

$U=&sum;_{x_i} U(x_i)p(x_i)$

Consider a Sequence of Lotteries $L_n$, Where $n$ is the Number of Clicks, E.G.

$L_1={(0.1,0) ,(0.9,1)}$

$L_2={0.2,0 ,0.8,2}$

$L_3={0.3,0 ,0.7,3}$

$L_4={0.4,0 ,0.6,4}$

Possible Expected Utilities in the Above Set of Lotteries

$U_1=0.9$

$U_2=1.6$

$U_3=2.1$

$U_4=2.4$

Example for Groups

$f(&pi;)$ = Rationality 

$q(&pi;)$ = Irrationality

$&pi;n$= lottery choice

$p(&pi;_n)=f(&pi;_n )+q(&pi;_n)$

$p(&pi;_n )=(number of people who chose decision)/(total number of people)$

Sum of all $q(&pi;)$ = 1

$q(&pi;_1 )=-q(&pi;_2 )$,

The same goes for $f(&pi;)$

In a study by Young et al. in 2012, it was found that the Risk-Taking Scale (RTS) correlated significantly with impulsivity and venturesomeness in the BART [5]. In a study by Benjamin and Robbins in 2007, sensation-seeking scores were significantly correlated with scores on the BART [6]. In a study by Skeel et al. in 2007, the BART final score (total money won) was significantly correlated with weekly alcohol consumption [7]. In a study by Lawyer in 2013, risk-taking in the BART was significantly associated with criminal behaviour [8]. In a study by Ko et al. in 2008, results were found which indicate that college students with internet addiction had a better performance on the Iowa gambling task [9].

Personality traits can be quantified and inputted into a risk-taking coefficient assessor.

Risk-taking coefficient for personality traits

$I$ = Impulsivity

$V$ = Venturesomeness

$a$ = weight of impulsivity

$b$ = weight of venturesomeness

$r=r(I,V)$

$=a&times;I+b&times;V$

In a study by Crisan et al. in 2009, it was found that there are significant effects of the serotonin-transporter-linked polymorphic region (5-HTTLPR) on social learning of fear, risk-taking and the framing bias in decision making, as well as on autonomic activity [10]. In a study by Bevilacqua and Goldman in 2013, it was found that impulsivity is a heritable, disease-associated trait, useful as an endophenotype for gene discovery [11]. In a study by Anokhin et al. in 2009, there was a significant increase in propensity for risk-taking from the age of 12 to 14, but even with changes into adulthood, individual differences remain relatively stable, as indicated by significant test-retest correlations [12].

Risk-taking coefficient for genetic traits

$c$ = weight of genetic traits

$G$ = Genetic traits that lead to risk-taking decisions

$r=r(G)$

$=c&times;G$

It has been noted that, if, in the process of mutual interactions between the members of the society, the amount of information of a decision-maker increases, then the attraction factor diminishes [13]. It was shown in a previous BART experiment that there was a good fit between the fixed effect model and the data as well as that the inclusion criteria applied to the studies yielded a homogeneous set of personality studies [4]. In another BART experiment, the adjusted average number of pumps on the blue balloon was higher for men than for women [4]. This same experiment looked into individual differences being correlated with personality traits, which are themselves stable upon repeat assessment. The participant’s age and ethnicity approached the conventional levels of statistical significance (Qs=2.47 and 2.60; dfs=1; ps=0.08 and 0.07), showing that the effect size trend tended to increase with participant’s age and with a greater representation of ethnic minorities. An RTS of the extent of the risk-taking behaviour was correlated negatively with social desirability and positively with impulsiveness and venturesomeness [5]. Whether someone has a high risk-taking coefficient will affect the attractiveness factor, they might view a potential winning as bigger than it really is.

Risk-taking coefficient model

$r$ = risk-taking coefficient

$I$ = Impulsivity

$V$ = Venturesomeness

$G$ = Genetic traits that lead to risk-taking 

$r=aI+bV+cG$

Weighting of Personality and Genetic Traits Model

$r$ = risk-taking coefficient

$&sum;_i a_i p_i$ = weighting of personality

$&sum;_i b_i g_i$ = weighting of genetics

$r=&sum;_i a_i p_i+&sum;_i b_i g_i$

Weighting Example

Where

$0.2I+0.8V$

Then their venturesomeness is four times their impulsivity.

**Figure 1:**
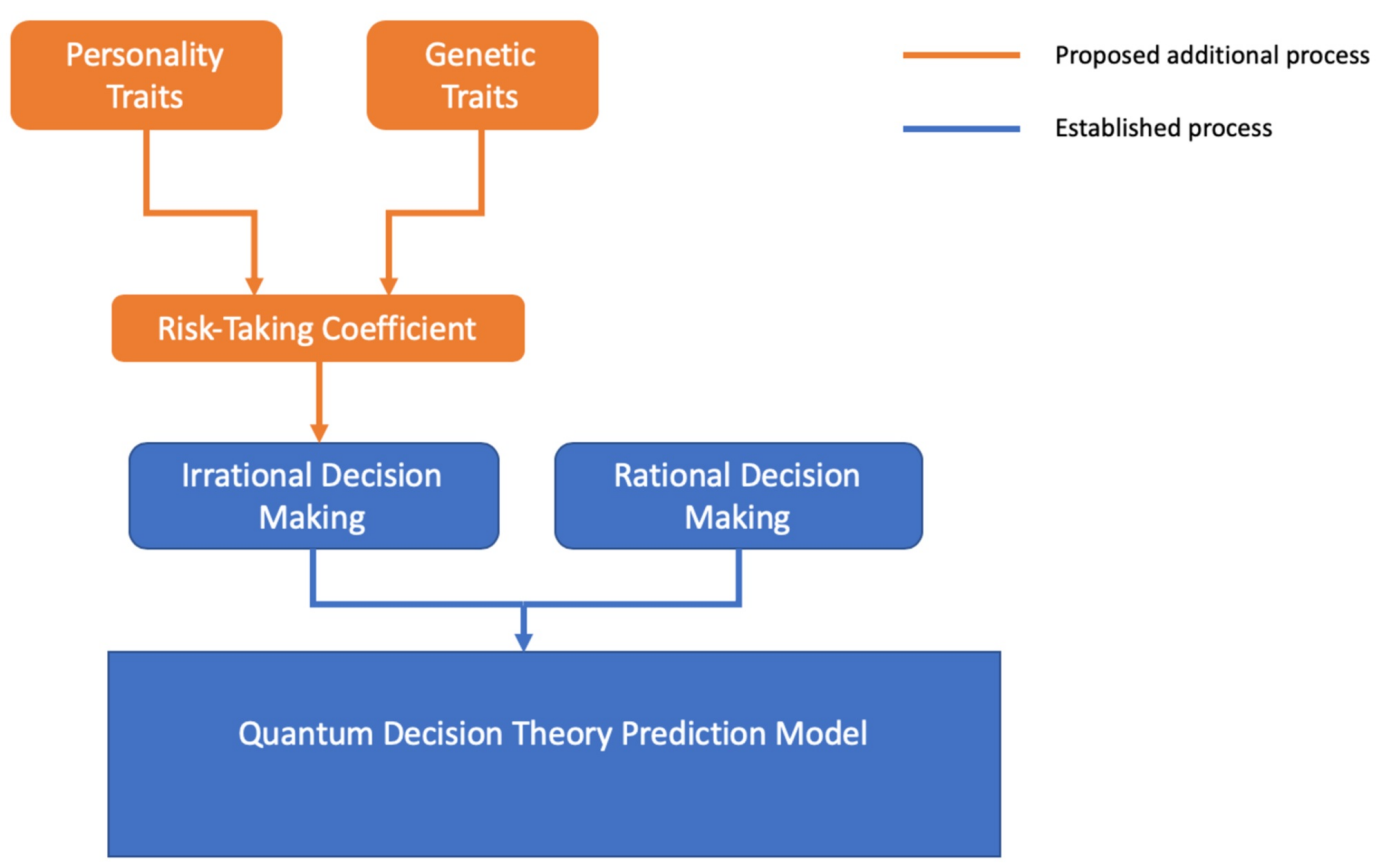
Diagram showing the process of irrational and rational decision making with the risk-taking coefficient, in the quantum decision theory prediction model

The diagram below in Figure 1 shows the process of irrational and rational decision making with the risk-taking coefficient, in the QDT prediction model.

Behavioural genetics, also referred to as behaviour genetics, is the field of scientific research that employs genetic methods to analyse and origins of various differences in behaviour. While the name “behavioural genetics'' implies the focus on biology influences, this environment generally investigates genetic and environmental influences, applying research patterns that provide removal of the confounding of genes and environment. Behavioural genetics was established as a scientific field by Francis Galton in the late 19th century. In the latter half of the 20th century, it saw renewed importance with research on inheritance of behaviour and mental disease in humans (typically using twin and family studies), also as research on genetically informative model organisms through selective breeding and crossings.

Mata et al. explained in 2012 that twin-studies suggest a significant portion of individual differences in the propensity to take risks resides in people’s genetic make-up, and there is evidence that variability in dopaminergic systems relates to individual differences in risky choice [14]. Dopaminergic means that it is releasing or involving dopamine as a neurotransmitter. They examined the link between risk-taking in BART and a variable number tandem repeat (VNTR) polymorphism in the 3′UTR of the dopamine transporter gene (SLC6A3/DAT1). A VNTR is a location in a genome where a short nucleotide sequence is organized as a tandem repeat. These can be found on many chromosomes, and often show variations in length (number of repeats) among individuals. The gene for DAT, which is the dopamine transporter, known as DAT1, is located on chromosome 5p15. The protein-encoding region of the gene is over 64 kb long and comprises 15 coding segments or exons. 

Secondary research has been performed for this article, using previous BART experiments and QDT research. Data for this research already exists, so only secondary research has been performed. Data collection has been sourced from several previous BART experiments and QDT articles. 

The personality trait correlations were positively correlated to risk-taking behaviour, which can be used to probabilistically predict decisions of individuals and groups in certain scenarios. These traits can be quantified and inputted into the prediction model created. The genetic trait correlations were positively correlated to risk-taking behaviour as well, which can be quantified and inputted into the prediction model at the same time as the personality traits. The personality traits and genetic traits can be used to assess the individual’s risk-taking coefficient and inputted into the QDT model created, to better predict decisions, as shown in Figure 1.

## Conclusions

Personality traits that are needed to predict the risk-taking decisions are specific to impulsiveness and venturesomeness, but also include criminality, alcohol consumption and sensation-seeking. Specific genetic traits can also be used to predict risk-taking decisions. Results from previous BART studies could be used to quantify personality and genetic traits, which with QDT, could be used to predict risk-taking behaviour and decisions. This research aimed to explore the ways in which these traits can be added to predictive models of risk-taking behaviour. The results in this research allow improved predictions, as well as further research in QDT. Genetics and personality traits can be used with QDT, to predict risky decisions. The risk-taking coefficient model can be used with the QDT prediction model, in order to better probabilistically predict decisions.
